# Observation Criteria for Physical Education Teachers to Identify Gifted Children through Invasion Games

**DOI:** 10.3390/ijerph16234830

**Published:** 2019-12-01

**Authors:** Alejandro Prieto-Ayuso, Juan Carlos Pastor-Vicedo, Sixto González-Víllora, Onofre Contreras-Jordán

**Affiliations:** 1 Department of Music, Arts and Physical Education Pedagogy, Faculty of Education, Camino Pozuelo s/n, University of Castilla-La Mancha, 16071 Cuenca, Spain; Alejandro.Prieto@uclm.es (A.P.-A.); Sixto.Gonzalez@uclm.es (S.G.-V.); 2 Albacete Balompié S.A.D., Prolongación de la Avenida de España s/n, 02006 Albacete, Spain; 3 Department of Music, Arts and Physical Education Pedagogy, Faculty of Education, Plaza de la Universidad 3, University of Castilla-La Mancha, 02001 Albacete, Spain; Onofre.CJordan@uclm.es

**Keywords:** gifted children, motor ability, talent identification, Physical Education, athletic talent

## Abstract

Whilst in other curriculum subjects, it exists observation criteria to detect gifted pupils, there is a paucity of information in Physical Education. For that reason, we aimed to reveal the observation criteria for identifying gifted pupils in Physical Education in an invasion game. Physical Education (PE) talent was evaluated combining results of questionnaire to experts, parents, and students. A validated nomination scale (NSIFT) and the Game Performance Evaluation Tool (GPET) were used. The research approach used in this study was transversal, descriptive, and inferential. The talent pool was composed of 18 gifted pupils aged 8–14 (M_age_ = 11.67, SD = 1.53). The results showed that the most discriminating criteria to identify PE talent were found to be precocity in both execution and decision-making in the acquisition of tactical principles and tactical-technical skills. Getting free was the skill that they best mastered. In conclusion, the importance of tactical principles and decision-making as observation criteria is emphasized. Future studies should focus on developing mentoring programs in Physical Education.

## 1. Introduction

Despite both the economic and social importance of reaching the sporting elite [1,2], the pathway to excellence, and the models of talent identification and development remain unclear [3]. This is mainly because the study of general athletic talent has proven to be complex due to the lack of reliability in instruments of evaluation, as well as the large number of terms inherent to the concept [4]. In that sense, Baker, Schorer, and Wattie [5] pointed some concerns related to talent identification (TI), going from the assumption that talent is a fixed capacity that can be identified early, the influence of talent beliefs on athletes development, the different levels of risk for talent selection decisions, biases evident in approaches to athlete selection, the inadequacy of current statistical approaches, the problems with using current performance to predict future outcomes, and how short-term priorities and competition between sports for talented athletes undermine the overall efficiency of athlete development systems. However, it has been clearly shown that activities in early sport participation play a key role in the development of sporting talent [6,7]. In that sense, two decades ago, Kirk and Gorely [8] pointed out that:

Physical education lessons provide the fundamental motor skills of running, throwing, jumping, kicking and so on, and these are then applied within an ascending scale of competitive contexts in inter-school, inter-district, inter-state and national sport.

*(p. 121)*

More recently, Bailey and Morley [9] studied the relation between natural talent and Physical Education (PE), highlighting that the implementation of programs in schools need to be built on a foundation of quality in general PE. As such, it seems to be clear that PE must be considered as an adequate starting point for identifying gifted children in sports [10,11,12], in the same way that these identification processes are conducted in other school subjects [13].

However, the way to detect Gifted and Talented (G&T) children in PE has not been the same. Traditionally, physical ability was the main indicator of talent, considering PE classes as forges of Olympic reserve [14]. In that sense, some authors have claimed a connection between PE and sports performance [8]. Currently, this conceptualization has been overtaken, understanding that the main goal of this subject must not be only TI, but also health [15,16]. Thus, the main objective of TI in PE is to give a wide range of opportunities to children to take part in sports during the child’s school life [17,18].

Since then, more researchers have highlighted how PE can contribute to that kind of process, from a cognitive perspective and not solely based on physical condition. For instance, Krombholz [19] investigated the development of physical characteristics, physical skills, and cognitive performance in preschool age, concluding that children with high motor performance at the beginning of the study did perform better in coordination, fitness, and manual dexterity compared to average or low performing children at each trial. Faber, Pion, Munivrana, Faber, and Nijhus-Van der Sanden [20] evaluated a perceptuomotor skills assessment as part of talent detection for table tennis with primary school children, highlighting that the assessment appears to be of added value for talent detection in table tennis at this young age. Santos, Jiménez, Sampaio, and Leite [21] designed the Skills4Genius sports-based training program based on thinking, motor skills, and in-game creative behavior in team sports. They tested the effects of the program with primary school children and affirmed that educators may apply this functional environment to inspire children’s disposition to move outside the box and trigger a creative spark in team sports players. Lovell, Bocking, Fransen, and Coutts [22] examined the factors influencing selection into playing levels and playing positions in a school-based soccer program, emphasizing a combination of maturation, anthropometry, physical capacity, technical ability, and motor competence in those gifted pupils. These results have important implications for talent development pathways in schools. Hoeboer et al. [23] examined the achievability and validity of an athletic skills track (AST) to assess fundamental movement skills among 6 to 12 year old children in a PE setting. The results indicated that the fundamental movement skills of these children can be assessed with a quick, convenient, and low-cost motor competence test in a physical education setting Nevertheless, in spite of the importance of PE lessons in sporting talent identification (TI), traditionally speaking, children have usually been exposed to traditional programs outside of class hours [24].

When it comes to detect sporting talent at an early age, coaches, parents, and teachers often find some difficulties [4,25]. These difficulties are related to inconsistencies in the definitions of “talent” and “sport” [26], as well as that the criteria for identifying them are mainly based on physical condition [27]. Contrarily to these approaches, new trends are championing other methods of evaluation for PE teachers such as measuring the tactical side of the pupil, rather than just the physical side [28], and evaluating the decision-making of young players in both sports and educational contexts. On one hand, in the sports context it is possible to highlight the works of González-Víllora, García-López, and Contreras-Jordán [29], who evaluated decision making and skill development in youth football players. They concluded that sports knowledge starts with a focus on attack and evolves to include defense knowledge. Práxedes, Moreno, Gil-Arias, Claver, and Del Villar [30] focused on the effect of two teaching programs, each utilizing modified games with varied levels of opposition, on decision-making in young players. They highlighted that that for groups with an average level of ability, training with increased numbers in attack provides players with an increased time to make better decisions and to improve their execution. More recently, Serra-Olivares, García-López, and Gonçalves [31] explored the effects of players’ level and age group on positional tactical behavior during 7- and 8-a-side football games. They concluded that most skilled and older players were more efficient in covering the available space, which may suggest greater tactical awareness.

On the other hand, in an educational context, Sánchez-Mora, García López, Del Valle-Díaz, and Solera-Martínez [32] gauged the declarative and procedural knowledge of the students as well as their performance in a modified invasion game (games in which the purpose is to invade the opponent’s territory while scoring points and keeping the opposing team’s points to a minimum, all within a certain time period). They claimed that determining tactical knowledge and performance by means of a modified invasion game is important in implementing an increase in the quality of the sessions and also the teaching programs. Moreover, linking tactical knowledge and performance to the game contexts that students have to resolve is thus an efficient way of investigating and identifying knowledge of the specific sports and students’ previous sporting experiences.

Finally, in order to link both contexts, García-López, González-Víllora, Gutiérrez, and Serra [33], designed and validated the Game Performance Evaluation Tool (GPET). It is an instrument that measures decision-making and the execution of technical-tactical actions in invasion games/sports. According to Griffin and Butler [34], the transferable skills can be passing an object, receiving an object, dodging, change of direction, traveling in multiple directions, speed and agility, spatial awareness, change of speed, anticipation, and footwork.

Thus, as understanding in one invasion game develops (i.e., in soccer), the performer is able to transfer knowledge (for example of which tactics to employ in a given situation) to another invasion game (i.e., field hockey) [35]. For that reason, and due to these kinds of games represent the most tactical complexity, invasion games are one of the best possibilities for evaluating gifted children, [36]. Nevertheless, although invasion games are being used for detecting gifted children in sports, PE teachers do not yet know what the observation criteria are that can serve as the basis for detecting and enhancing sporting talent [37].

In brief, the concept of talent in sport has changed its meaning, from a unidimensional conceptualization (physical) to a global concept in which not only the physical parameters are important, but also decision-making, technical skills and psychological aspects such as motivation. Some decades ago, the studies related to game performance in PE began to be focused on PE context; however, not so with gifted students. Thus, while teachers have criteria to detect gifted pupils in other curriculum areas, there is a gap in PE. As such, with the purpose of helping PE teachers to identify gifted pupils, this work is aimed at revealing the observation criteria for identifying gifted pupils in PE, as well as to reveal the relation between decision-making and skill execution of gifted pupils. Thus, firstly, we analyzed the game performance of 18 gifted pupils selected from a group of 201 participants aged 8–14 years in invasion games. Secondly, we determined which skill defined them most. We hypothesized that tactical-technical skills can be useful as observation criteria for PE teachers in their classes (Hypothesis 1). Secondly, we hypothesized a strong relation between decision-making and technical skills in those pupils nominated as talented (Hypothesis 2).

## 2. Materials and Methods

### 3.1. Study Design

The design of the study was descriptive, inferential, and transversal [38]. We aimed to describe a group of PE pupils. However, after analyzing the data inferentially, we could establish conclusions applicable to the population. Finally, the study was transversal because it was conducted in a specific moment of the time.

### 3.2. Participants

Initially, a total of 201 Spanish PE pupils aged 8–14 took part in the study. All participants practiced an invasion game (football) in extracurricular hours. Thus, we ensure both a good previous knowledge in invasion games and commitment to practice [39]. According to Françoys Gagné, the top 10% of a population has talent. As such, only those among the top 10% of our group were nominated as gifted by the nomination scale of Prieto-Ayuso, Pastor-Vicedo, and Contreras-Jordán [40] were included in the talent pool. Thus, a total of 18 PE pupils formed the final sample (M_age_ = 11.6, SD_age_ = 1.5; M_weight_ = 42.3 kg, SD_weight_ = 10.6 kg; M_height_ = 151 cm, SD_height_ = 12 cm). The game performance of these gifted players was analyzed.

As sample size was composed of underage children, parents gave their consent to participate in the study, whereas children gave their verbal approval prior to data collection. The research project was fully approved by the Ethical Committee of the University of Castilla-La Mancha. The research has been developed under the recommendations of the Declaration of Helsinki.

### 3.3. Instuments

First, the Nomination Scale for Identifying Football Talent (NSIFT) of Prieto-Ayuso et al. [40] was used to select those gifted pupils. It evaluates three dimensions: (a) cognitive, (b) psychological, and (c) motivation. The scale is a 13 item questionnaire:

1. Interprets the instructions correctly;
2. Usually anticipates play;
3. Generally makes the right decision;
4. Executes skills very quickly;
5. Able to read the game clearly and quickly;
6. Has good positional sense;
7. Knows where their teammates are on the pitch;
8. Makes an effort in matches and training;
9. Keen to learn and develop;
10. Able to concentrate in matches and/or training;
11. Possesses a winning mentality;
12. Has a positive attitude;
13. Willing to take on responsibilities.

There are no negative items in the scale. It was administered to the stakeholders: players, parents, and teachers. Players evaluated their peers, parents evaluated their children, and teachers individually evaluated their pupils. Based on the research conducted by Rogers [41] in an educational context, on the one hand, parents and teachers evaluated through a Likert scale, from 1 “very disagree” and 5 “very agree”. The highest score was 65 points and the lowest was 12 points. On the other hand, pupils evaluated their classmates, nominating who was the best in each item. The triangulation of the information derived from parents, teachers, and players showed which were the most gifted pupils. The internal consistency of NSIFT among the stakeholders for this research scored 0.85.

Secondly, the Game Performance Evaluation Tool (GPET) assessed decision-making and technical skills [33]. The evaluation is done at two different levels. The first level evaluates tactical understanding: keeping possession of the ball (1A), advancing towards the opponent’s goal (2A), and scoring a goal (3A). At the second level, GPET separates the cognitive components from the decision-making and technical skills, such as control, passing, dribbling, driving, and getting free.

To evaluate decision-making, a value of 1 was assigned to appropriate decisions and 0 to inappropriate decisions. An appropriate decision was defined as when the game-situation principle and the principle applied by the player was the same in a decision-making unit (DMU), defined as the technical-tactical actions made by an attacking player in each play (e.g., passing the ball, running forward, keeping the ball, etc.). If they were not the same, then the decision-making was deemed inappropriate. Likewise, to evaluate execution, a value of 1 was assigned to successful executions and 0 to unsuccessful executions. The total number of these decisions and executions was divided by the sum of the total number of decisions and executions, and multiplied by 100.

### 3.4. Procedures

First, NSIFT was used to select the gifted pupils. The main researcher got ethical approval from the university and families. The approval of the Ethical Committee was published on the report with the reference code 03/2016. The main researcher was placed in a room with adequate conditions to apply the questionnaires. The pupils completed the scale one by one with the main researcher. It took three weeks to have all the pupils’ questionnaires completed. The pupils evaluated their peers. The experts completed the scale at the end of their daily schedule; the main researcher gave the questionnaires to them, they read and resolved any doubts, and then completed the scale. Teachers evaluated their pupils one by one. It took one week to have all the teachers’ questionnaires completed. Finally, the main researcher met with the parents of each age group. They read the scale, resolved any doubts, and completed the questionnaires. Parents evaluated their children. It took three weeks until all the parents’ questionnaires were completed. No similar previous studies have conducted an identification process as rigorous as this [30,32,42,43].

Secondly, the invasion games (football matches) were divided into three age groups: U-10 (9–10 years old), U-12 (11–12 years old) and U-14 (13–24 years old). The grass of the field was artificial. The number of players in U-10 and U-12 was 8 vs. 8 (45 × 30 m) and in U-14 was 11 vs. 11 (90 × 60 m). Although the entire sample (*n* = 201) took part in the invasion game, only those children selected were analysed (*n* = 18). When the talent pool was formed, six full matches randomly chosen from the official league in each age group, were observed. The main researcher travelled to where the matches took place, due to the matches taking place in extracurricular hours, with the aim of maintaining the ecological dynamic of the study. Due to the schedule’s needs, these matches took place at the end of the season and they were all recorded in one month, from the first until the last match observed. We used a Sony HDR-AS100VR Action Cam Full HD camera, DSK-Sony battery, Samsung memory card, and Cullmann tripod. The camera was located in the highest accessible point in which each match took place, in order to follow the GPET guidelines of recording the matches in the foreground. After recording the matches, they were uploaded to a HP 6730 s computer. Then, the first ten minutes of the matches were analyzed according to the GPET. The GPET sheet was used for analyzing the matches, and this information was written in IBM SPSS Statistics, version 24.0 (IBM, Armonk, NY, USA). Once the data were included in SPSS, the analysis was conducted.

Thirdly, the main researcher was required to have four sessions of training before using GPET. In the first session, a co-author of GPET gave a theoretical lesson about the instrument. In the second session, the main researcher raised any issues with same co-author after having studied how to code each action. In the third session, the main researcher analyzed a video of three children playing invasion games, and completed the GPET log sheets. The analysis was repeated one week later. Finally, in the fourth session, the main researcher calculated, on the one hand, the intraobserver reliability. On the other hand, the interobserver reliability was calculated with the co-author of the GPET. They both scored a superior coefficient of 0.8.

### 3.5. Statistical Analysis

The analysis was done in the three age groups: U-10, U-12, and U-14. A descriptive analysis was used for the nature of the game through mean (M), standard deviation (SD), frequencies, and percentages. Then, an inferential analysis was conducted. Non-parametric tests were used, considering the size of the talent pool (*n* = 18), as was the Shapiro–Wilk test (*p* < 0.05). According to the design of the study, the Kruskal–Wallis test, which is used for comparing two or more independent samples of equal or different sample sizes, was used to find the differences between the game-situation principle and the game-play principle in each age group. The Mann–Whitney U test, which is used when there are only two groups, was used to establish the differences between both the tactical principles and technical elements in each pair of age groups. *p* values were accompanied with metrics such as effect sizes (*d*), considering as trivial (0–0.19), small (0.20–0.49), medium (0.50–0.79), and large (0.80 and greater), defined as [44]:

(1) $$d=\frac{M1-M2}{\sqrt{(S^{2}1+S^{2}2)/2}}$$

Finally, a correlation analysis (r) between decision-making and skill execution was performed using Spearman’s rho. The statistical program used was SPSS v. 24.0. The confidence level was 95%.

## 3. Results

Regarding the nature of the game, a total of 771 DMUs were completed by the 18 gifted pupils selected. Concerning the 1A tactical principle, 250 DMUs were recorded (32% of the total), of which 87.2% were completed correctly. In the 2A tactical principle, 509 DMUs were completed (65.2% of the total), of which 94.1% were completed correctly. The smallest number of DMUs were recorded in the 3A tactical principle. The total number of DMUs was 12 (1.5%), all of which were completed correctly.

The 1A tactical principle increased through the age groups (26.8% in U-10, 34.3% in U-12, and 34.7% in U-14). However, it was not repeated in the 2A tactical principle, decreasing through the age groups (72.1% in U-10, 63.7% in U-12s, and 63.5% in U-14s). In relation to the 3A tactical principle, the highest score was obtained in the U-12 age group (1.8%), making it the most effective. In contrast, the principle barely existed in the U-10 age group (0.9%). It also decreased in the U-14 age group (1.6%) (see Table 1).

Table 2 reveals the existence of significant differences in the 2A tactical principle, in relation to correct decisions and in the overall relationships of the principles of situation and application with correct decision-making and the successful execution of these decisions.

Regarding tactical principles (see Table 3), the results revealed significant differences in tactical principles between the U-10 and U-12 age groups, and the U-12 and U-14 age groups, which was not the case between the U-10 and U-14 age groups. Furthermore, the tactical principle that most frequently appeared was 2A, followed by the other two tactical principles (1A and 3A).

Regarding technical elements analysis (see Table 4), the most significant differences appeared in the getting free skill, and in both decision-making and execution (U-10 and U-12, and U-12 and U-14). The significant differences in relation to getting free can be seen not only in the overall result, but also in decision-making and in successful execution in the two pairs of age groups (U-10 and U-12, and U-12 and U-14). Regarding tactical principles, the most repeated was 1A, which was presented in the U-10 and U-12, and the U-10 and U-14 pairs of age groups. The second principle most repeated was 2A, in the U-10 and U-12 age-group pairs.

In relation to the attacking pupil on the ball, there are various technical elements with significant differences. In terms of moving with the ball, significant differences were shown in the U-10 and U-12 age-group pair, related to both execution and decision-making. On the other hand, passing was a technical element that arose in the two pairs of age groups (U-10 and U-12, and U-12 and U-14), being related more often to decision-making instead of execution. There were no significant differences in shooting in any of the three pairs of age groups.

Table 5 shows the variables in which significant differences were found according to Spearman’s rho. The results showed that passing and getting free are the technical elements with the highest number of positive correlations, both tactical principles 1A and 2A. It can also be noted that the principle with the most positive correlations was 1A, followed by 2A. The most repeated technical elements were passing and getting free. Finally, regarding shooting in 3A, there is a positive correlation between decision-making and execution in U-12 and U-14.

## 4. Discussion

The purpose of the work was to reveal the observation criteria for identifying gifted pupils in PE, as well as to reveal the relation between decision-making and skill execution of gifted pupils. The main findings show a greater frequency and effectiveness in the 2A tactical principle, followed by 1A and 3A. Moreover, the getting free skill was the technical element which showed the most significant differences in game performance in the three age groups analyzed. Finally, the correlation analysis between decision-making and technical execution revealed positive and significant differences.

### 4.1. Analysis of Effectiveness in Tactical Principles and Technical Skills

TI in sports has been a common topic over the last decades [45]. This study showed knowledge about TI in a school context, due to the lack of previous studies which focused on that [9].

Thus, the results point a greater frequency and effectiveness in the 2A tactical principle, followed by 1A and 3A, similarly to the results obtained by González-Víllora et al. [29] previously. However, the effectiveness increased in 1A (from 88.13% to 94.10%) and 3A (from 84.40% to 100%). The 2A principle showed similar values (from 87.75% to 87.20%). This may be due to the identification process that was conducted previously. Otherwise, the game performance between talented and non-talented can be similar [30].

In the U-10 age group, the effectiveness was also higher in this study than previous ones [46] in tactical principles and technical elements. In the U-12 age group, the study here presented higher values again in comparison with the work of González-Víllora et al. [47]. These results can be explained because this study was conducted previously, and was a rigorous TI process through the NSIFT [40]. Thus, it is possible to show the importance of applying a reliable instrument for selecting the talent pool of the sample. Although the coaches’ view is important for this kind of processes, it has been recently demonstrated that it must be combined with other objective tools [48,49] in order to approach the selection to a better process of TI.

Unlike U-10, higher values were also recorded for the technical elements in U-12, in decision-making in passing, as well as technical execution. The same results were found in decision-making in moving with the ball, decision-making in the getting free skill, and in the execution of this technical element. Thus, according to Práxedes et al. [30], for helping to young players with a low level of sporting skill, it is essential to carry out situations involving less tactical complexity (e.g., 3 vs. 1 or 4 vs. 2) in order to favor adequate learning. Having these results in mind, PE teachers can use invasion games for this due to the fact that these kind of games are able to measure not only decision-making, but also skill execution, avoiding the rejection of gifted children by maturity biases [36].

On the other hand, in the U-14 age group, the use of tactical principles is similar in comparison with the results obtained in other studies, for instance, González-Víllora et al. [29,50]. The 1A tactical principle increased throughout all age groups. Meanwhile, 2A and 3A decreased. The study therefore confirms the previous results in which the development in game performance evolves from the 2A to the 1A tactical principle. As such, it might be deduced that young players try to score as quickly as possible, but as they move through the age groups, they play with greater depth and width. In accordance with these results, previous studies revealed an excessive use of dribbling in U-8 and U-10 [46], and a greater number of passes and less dribbling in U-12 and U-14. These results were also shown in the study of González-Víllora et al. [47].

The high effectivity of all these elements (more than 80%) means that previous studies could hold a bias in the TI process [51], because this process has been exclusively based on technical skills like, for instance, the Loughborough Soccer Passing Test, whose reliability remains unclear [52].

### 4.2. Relationship and Differences between Decision-Making and Technical Skills

The correlation analysis between decision-making and technical execution also revealed positive and significant differences, according to what Blomqvist, Vänttinen, and Luhtanen [53] found a decade ago. It might help PE teachers to get a better understanding about the skill acquisition of their pupils by taking up new pedagogical contents, like those educational experiences [54,55].

The getting free skill was the technical element which showed the most significant differences in game performance in the three age groups analyzed, according to other results in U-10 [29]. It can be stated in terms of tactical-technical principles that there was a greater level of effectiveness in U-14, which may be explained by the ability of the players (Bailey et al., 2004). Based on Sánchez-Mora et al. [32], the students had difficulties in using their tactical knowledge in gameplay in the initial stages of learning the game. However, it should also to be pointed out that effectiveness in execution is acquired at an earlier stage in the sample used in this study than in previous studies, especially in 1A [29].

The results obtained will provide great information for PE teachers in a school context. First, they will have a better practical knowledge of observation criteria when identifying gifted pupils, which are unknown so far in a school context. Secondly, PE teachers will be aware of the overall tactical-technical performance in invasion games of gifted pupils in each age group. These will help teachers to test the good or bad performance of each pupil when using, for example, the Skills4Genius training program [21] or the school-based soccer program [22]. Thus, these results continue a line of investigation which provides instruments and programs to provide PE teachers with tools to use in class. In that sense, it is essential to maintain research in this line of investigation, with the purpose to improve the teaching–learning process in sports focused on gifted children. So far, those children with special needs have been the focus of researchers (inclusive education) in PE [56], and there is a scarcity of researching about how to deal with gifted children in PE [9]. Traditionally, these children have been trained in a traditional way for both PE teachers and researchers [24]. Nevertheless, new trends are pushing other evaluation methods focused on a multidimensional evaluation [12] with invasion games as a suitable way to conduct this process in a school context [36].

Thus, as research prospect, a noteworthy future line of investigation is the need to continue developing this study through older age groups. Moreover, PE teachers must put mentoring programs into practice with these gifted students. It is also necessary to include a female sample, asking whether their overall performance is dissimilar to a male sample. Finally, future studies should focus on what criteria PE teachers can follow in other kinds of games in PE.

## 5. Conclusions

This study highlights the existence of reliable observation criteria for identifying gifted pupils through invasion games in PE. Thus, both tactical principles and decision-making must be taken into account by coaches and PE teachers as identification criteria for gifted pupils (Hypothesis 1). Moreover, the strong relationship shown between decision-making and technical skills can help PE teachers to conduct this kind of process (Hypothesis 2). As such, they will have available these criteria as a measurement of gifted pupils, which must be accompanied by the stakeholders’ opinions (coaches, parents, teachers). Previous studies have focused the identification of gifted students in other curriculum areas, but not in PE. However, this study reveals some criteria that can be used by PE teachers in their classes, unknown until now. The methodology applied (parents, peers, and teacher opinions) has not been used in a sporting context previously, according to the literature reviewed. Out of all tactical principles and tactical-technical skills aforementioned, the getting free skill was the most decisive aspect among gifted pupils.

Moreover, the results suggest that this kind of identification process can also be made in different sports branches apart from soccer, and can be applied to invasion games in general, as they share tactical principles. For that reason, this study is very useful as PE teachers teach many sports (including invasion games) within their curricula.

## Figures and Tables

**Table 1:** Percentage of effectiveness (%) of tactical principles and tactical-technical skills of gifted pupils (*n* = 18).

			U-10 (%)	U-12 (%)	U-14 (%)
Control the ball			95	95	90
Tactical principles					
1A (Maintaining)			82.4	87.3	90.2
2A (Attacking)			89.5	96.1	96
3A (Scoring)			100	100	100
Tactical-technical skills					
Attacker on Ball	Passing	DM	81.2	87.9	93.7
		Ex	71.8	74.1	84.3
	Dribbling	DM	40	70.5	83.3
		Ex	40	70.5	75
	Shooting	DM	-	85.7	100
		Ex	-	42.8	66.6
Attacker off Ball	Getting free	DM	93.1	94.9	96.2
		Ex	85.2	90.4	97.2
	Support	DM	90.4	90.1	97.4
		Ex	93.6	81.9	84.8

DM: decision making; Ex: execution.

**Table 2:** Kruskal–Wallis test and effect size (*d*) between tactical principles (*n* = 18).

	Chi-Square	Sig.	*n* ^2^	*d*
PpSitApl1A1A	4.87	0.087	0.137	0.796
PpSitApl1A2o3A	1.08	0.581	−0.044	NaN
PpSitApl1Ano	1.06	0.588	−0.045	NaN
PpSitApl2A2A	9.52	0.009 **	0.358	1.494
PpSitApl2A1o3A	0.20	0.901	−0.086	NaN
PpSitApl2Ano	0.94	0.624	−0.05	NaN
PpSitApl3A3A	0.97	0.616	−0.049	NaN
PpSitApl3A1o2A	0.00	1.00	−0.095	NaN
PpSitApl3Ano	0.00	1.00	−0.095	NaN
PpSit1A	4.25	0.119	0.107	0.693
PpSit2A	6.93	0.031 *	0.235	0.108
PpSit3A	0.97	0.616	−0.049	NaN
PpSitAplTotal	7.85	0.020 *	0.279	0.243
PpSitAplTotalEx	8.87	0.012 *	0.327	0.395

** *p* < 0.01, * *p* < 0.05. Sig = *p*-Valor; Pp = principle; Sit = situation; Apl = application; 1A = 1st principle: maintaining; 2A = 2nd principle: attacking; 3A = 3rd principle: scoring; Ex = success; *n*^2^ = Eta squared; *d* = d_Cohen_; NaN = Not a number.

**Table 3:** Mann–Whitney U test in tactical principles and effect size (*d*) between tactical principles (*n* = 18).

Pair of Categories Analysed	Tactical Principles	U-Mann Whitney		*n* ^2^	*d*
		Sig. Asymptotic (Two-Sided)	Sig. Exact [2*(Sig. One-Sided)]		
U-10 with U-12	Principle Sit 1A 1A	0.054	0.065	0.308	1.333
	Principle Sit 2A 2A	0.004	0.002	0.692	3
	Principle Sit 2A	0.024	0.026	0.026	0.328
	Principle Sit Apl Tot Td	0.004	0.002	0.692	3
	Principle Sit Apl Tot Ex	0.004	0.002	0.692	3
U-10 with U-14	-	-	-	-	-
U-12 with U-14	Principle Sit 2A 2A	0.019	0.150	0.449	1.806
	Principle Sit 2A	0.024	0.026	0.001	0.046
	Principle Sit Apl Tot Ex	0.054	0.065	0.308	1.333

Sig = *p*-Valor; Pp = principle; Sit = situation; Apl = application; 1A = 1st principle: maintaining; 2A = 2nd principle: attacking; 3A = 3rd principle: scoring; Ex = success; Tot = total; *n*^2^ = Eta squared; *d* = d_Cohen_.

**Table 4:** Mann–Whitney U in technical tactical skills and effect size (*d*) (*n* = 18).

Pair of Categories Analysed	Technical-Tactical Skills	U-Mann Whitney		*n* ^2^	*d*
		Sig. Asymptotic (Two-Sided)	Sig. Exact [2*(Sig. One-Sided)]		
U-10 with U-12	Passing DM 1A	0.012	0.009	0.513	2.054
	Passing Ex 1A	0.018	0.015	0.449	1.806
	Getting free DM 1A	0.024	0.026	0.419	1.698
	Getting free Ex 1A	0.020	0.015	0.449	1.806
	Dribbling Ex 2A	0.006	0.009	0.547	2.198
	Getting free Ex 2A	0.036	0.041	0.361	1.504
	Getting free DM Total	0.016	0.015	0.481	1.925
	Dribbling Ex Total	0.008	0.009	0.547	2.198
	Getting free Ex Total	0.004	0.002	0.692	3
U-10 with U-14	Getting free Ex 1A	0.037	0.041	0.361	1.504
U-12 with U-14	Control Ex	0.009	0.009	0.547	2.198
	Passing DM total	0.054	0.065	0.308	1.333
	Getting free DM total	0.024	0.026	0.419	1.698
	Getting free Ex total	0.045	0.041	0.334	1.416

Sig = *p*-Valor; DM = Decisión-Making; Ex = execution; 1A = 1st principle: maintaining; 2A = 2nd principle: attacking; *n*^2^ = Eta squared; *d* = d_Cohen_.

**Table 5:** Significant correlations (*p* < 0.05; Spearman’s Rho) in tactical-technical skills between decision-making and execution (*n* = 18).

Age Group	Tactical-Technical Skill	Tactical Principles	*r*	*p*
U-10	Passing	1A	0.914	0.011
	Getting free	1A	0.955	0.003
	Support	1A	1.00	0.000
	Passing	2A	0.922	0.009
	Getting free	2A	0.978	0.001
	Support	2A	0.953	0.003
U-12	Passing	1A	0.900	0.015
	Getting free	1A	0.954	0.003
	Support	1A	0.942	0.005
	Passing	2A	0.869	0.025
	Dribbling	2A	0.974	0.001
	Getting free	2A	0.982	0.000
	Support	2A	0.899	0.015
	Shooting	3A	0.945	0.004
U-14	Passing	1A	0.998	0.000
	Dribbling	1A	1.00	0.000
	Getting free	1A	0.974	0.001
	Support	1A	0.866	0.026
	Passing	2A	0.975	0.001
	Dribbling	2A	0.963	0.002
	Getting free	2A	0.998	0.000
	Support	2A	0.965	0.002
	Shooting	3A	0.926	0.008

1A = 1st principle: maintaining; 2A = 2nd principle: attacking; 3A = 3rd principle: scoring; *r* = correlation coefficient; *p* = *p*-Valor.
